# Healthcare providers' expected barriers and facilitators to the implementation of person‐centered long‐term follow‐up care for childhood cancer survivors: A PanCareFollowUp study

**DOI:** 10.1002/cam4.70225

**Published:** 2024-10-23

**Authors:** Dionne Breij, Lars Hjorth, Eline Bouwman, Iris Walraven, Tomas Kepak, Katerina Kepakova, Riccardo Haupt, Monica Muraca, Irene Göttgens, Iridi Stollman, Jeanette Falck Winther, Anita Kienesberger, Hannah Gsell, Gisela Michel, Nicole Blijlevens, Saskia M. F. Pluijm, Katharina Roser, Roderick Skinner, Marleen Renard, Anne Uyttebroeck, Cecilia Follin, Helena J. H. van der Pal, Leontien C. M. Kremer, Jaqueline Loonen, Rosella Hermens, the PanCareFollowUp Consortium

**Affiliations:** ^1^ Departments of Hematology and IQ Health Radboud University Medical Centre Nijmegen The Netherlands; ^2^ Pediatrics, Department of Clinical Sciences Lund Lund University, Skåne University Hospital Lund Sweden; ^3^ International Clinical Research Centre (FNUSA‐ICRC) at St. Anne's University Hospital Masaryk University Brno The Czech Republic; ^4^ Epidemiology and Biostatistics Unit and DOPO Clinic IRCCS Istituto Giannina Gaslini Genoa Italy; ^5^ Childhood Cancer Research Group Danish Cancer Society Research Centre Copenhagen Denmark; ^6^ Department of Clinical Medicine, Faculty of Health Aarhus University and Aarhus University Hospital Aarhus Denmark; ^7^ Childhood Cancer International Europe Vienna Austria; ^8^ Faculty of Health Sciences and Medicine University of Lucerne Lucerne Switzerland; ^9^ Princess Máxima Centre for Pediatric Oncology Utrecht The Netherlands; ^10^ Faculty of Medical Sciences Newcastle University, Royal Victoria Infirmary (Sir James Spence Institute) Newcastle UK; ^11^ Great North Children's Hospital Newcastle UK; ^12^ Departments of Pediatric Hematology and Oncology, and Oncology University Hospitals Leuven Leuven Belgium

**Keywords:** barriers and facilitators, cancer survivorship, childhood cancer, follow‐up care, healthcare providers, implementation science, late effects

## Abstract

**Background:**

Childhood cancer survivors face high risks of adverse late health effects. Long‐term follow‐up care for childhood cancer survivors is crucial to improve their health and quality of life. However, implementation remains a challenge. To support implementation of high‐quality long‐term follow‐up care, we explored expected barriers and facilitators for establishing this follow‐up care among healthcare providers from four European clinics.

**Methods:**

A qualitative study was conducted using four focus groups comprising 30 healthcare providers in total. The semi‐structured interview guide was developed based on the Grol and Wensing framework. Data was analyzed following a thematic analysis, combining both inductive and deductive approaches to identify barriers and facilitators across the six levels of Grol and Wensing: innovation, professional, patient, social, organizational and economic and political.

**Results:**

Most barriers were identified on the organizational level, including insufficient staff, time, capacity and psychosocial support. Other main barriers included limited knowledge of late effects among healthcare providers outside the long‐term follow‐up care team, inability of some survivors to complete the survivor questionnaire and financial resources. Main facilitators included motivated healthcare providers and survivors, a skilled hospital team, collaborations with important stakeholders like general practitioners, and psychosocial care facilities, utilization of the international collaboration and reporting long‐term follow‐up care results to convince hospital managers.

**Conclusion:**

This study identified several factors for successful implementation of long‐term follow‐up care for childhood cancer survivors. Our findings showed that specific attention should be given to knowledge, capacity, and financial issues, along with addressing psychosocial issues of survivors.

## Graphical abstract

This study identified barriers and facilitators for successful implementation of long‐term follow‐up care for childhood cancer survivors. Our findings showed that specific attention should be given to knowledge, capacity and financial issues, along with addressing psychosocial issues of survivors.

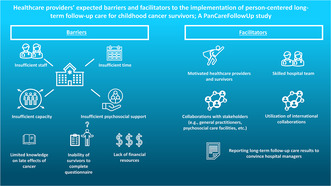

## 1 INTRODUCTION

The number of childhood cancer survivors (CCSs) is increasing. Currently, the estimated population of CCSs in Europe is approximately 500.000.^2^ CCSs face a high risk of developing adverse late health effects due to their cancer history and treatment. These late effects are heterogeneous, occurring on the physical, psychological, and social level^3^, ^4^, ^5^, ^6^, ^7^, ^8^, ^9^, ^10^, ^11^, ^12^, ^13^, ^14^, ^15^ and lead to higher morbidity and mortality rates compared to age and sex‐matched controls.^12^, ^16^, ^17^, ^18^, ^19^, ^20^, ^21^ The quality of life of CCSs is often affected by late effects,^4^, ^5^, ^6^, ^7^, ^17^ emphasizing the necessity for long‐term follow‐up care (LTFU) to improve CCSs' health and quality of life.^6^, ^22^, ^23^

Due to the heterogeneity in incidence, type, and severity of late effects, a person‐centered multidisciplinary care model is necessary to guide the organization of LTFU care for CCSs.^24^ High‐quality LTFU care is based on evidence‐based guidelines for screening and surveillance of late health effects after cancer treatment and person‐centered care.^22^, ^25^, ^26^, ^27^, ^28^, ^29^ Despite the available literature on evidence‐based (models of) LTFU care, sustainable implementation remains a challenge.^30^, ^31^, ^32^, ^33^, ^34^ The majority of the European CCSs still has limited access to high‐quality LTFU care.^35^

To enhance implementation of LTFU care for CCSs in Europe, the PanCareFollowUp (PCFU) consortium, established in 2018, developed the PCFU Care intervention based on a Dutch LTFU care model.^1^, ^24^ The overall aim of the intervention is to empower childhood cancer survivors across Europe and to improve their health and quality of life by providing person‐centered survivorship care.^1^ The PCFU Care intervention will be evaluated through a prospective cohort study conducted at four pediatric cancer‐focused LTFU care clinics, each representing different healthcare systems with varying levels of pre‐existing survivorship care implementation.^1^, ^36^

However, most innovations do not implement themselves. Tailored implementation strategies have the potential to improve implementation efforts.^37^ Identifying barriers and facilitators is a critical first step in developing an effective implementation strategy.^38^ Existing reviews on barriers and facilitators for implementing LTFU care for cancer survivors mainly focused on adult cancer survivors and specific cancer types.^33^, ^34^ Barriers and facilitators are insufficiently studied in the context of establishing LTFU care for the heterogenous population of CCSs. Therefore, we performed a pre‐implementation study aiming to explore expected barriers and facilitators for the implementation of the PCFU Care intervention among health care providers (HCP) involved in LTFU care for CCSs in four European clinics.

## 2 METHODS

### 2.1 Study design and setting

A qualitative study was performed using semi‐structured focus groups with HCPs to explore potential barriers and facilitators for the implementation of the PCFU Care intervention. This study followed the consolidated criteria for reporting qualitative research (COREQ checklist)^39^ and adhered to local medical ethical standards of the participating centers.

As part of the European‐wide PCFU project (Horizon 2020 grant), this study included four European LTFU care clinics for CCSs, located in Belgium, the Czech Republic, Sweden and Italy.

### 2.2 PCFU Care intervention

**FIGURE 1:**
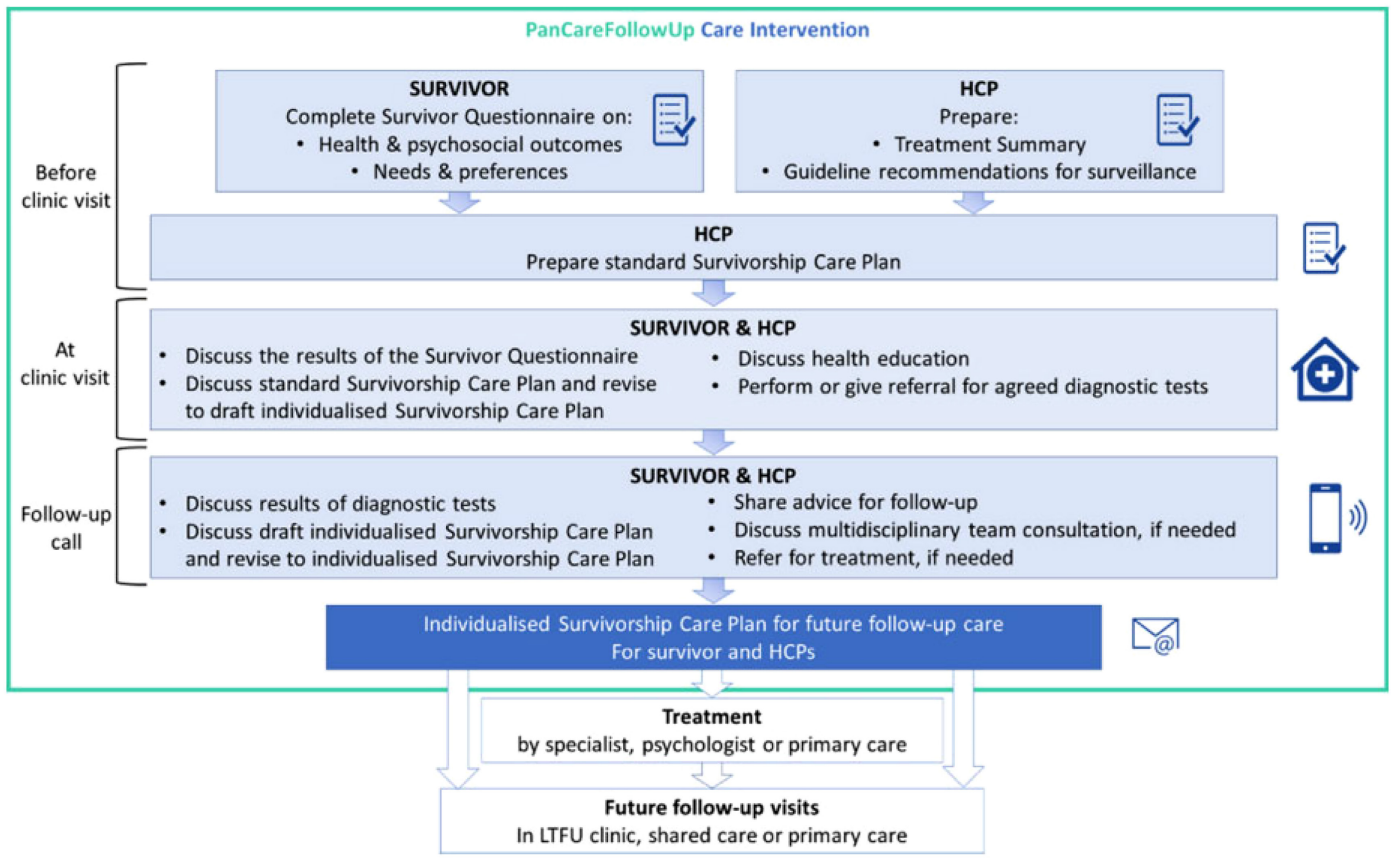
PanCareFollowUp Care intervention; developed by the PCFU consortium.^1^

The PCFU Care intervention is based on international guidelines for surveillance on late effects and person‐centered care. The organizational structure for person‐centered is based on the pillars of Eckman et al.^25^ and consist of three phases; initiating, integrating and safeguarding a partnership between patients and HCPs. The first phase of the PCFU Care Intervention, involving the initiation of a partnership between CCSs and HCPs, takes place before the clinic visit. During this phase, both CCSs and HCPs prepare the clinic visit by completing a questionnaire (the survivor questionnaire) and a treatment summary, respectively. The survivor questionnaire is web‐based and gathers information about the CCSs' health, well‐being, medication use, medical and family history, lifestyle, social situation, healthcare needs, and preferences for care with their HCP.^1^ The second phase, concerning integration of this established partnership between CCSs and HCPs, involves discussing the CCSs' health and follow‐up care based on shared‐decision making, during the clinic visit. Lastly, this partnership is safeguarded by a follow‐up call during which the results of diagnostic tests and recommendations for further follow‐up care are discussed. These results and recommendations are summarized in a survivorship care plan. Figure 1 shows the important steps within these three phases. The development and features of the PCFU Care intervention are described elsewhere.^1^

### 2.3 Study population

A purposive sampling strategy was applied to recruit participants for this study. At each of the four centers where the PCFU Care intervention will be tested for feasibility, we aimed to organize one focus group with a minimum of five HCPs per group. HCPs from the participating clinics involved in LTFU care were invited by local representatives of the PCFU project. HCPs that were willing to participate registered via e‐mail.

### 2.4 Data collection

On‐site focus groups were conducted in Belgium, the Czech Republic, and Italy between September 2019 and November 2019. Due to pragmatic reasons, a video conference application was used for Sweden. Focus groups in Sweden and the Czech Republic were conducted in English and focus groups in Belgium and Italy were conducted in their native language. Two independent experts in qualitative research and implementation research conducted the focus groups, with a note‐taker present. Prior to the focus groups, participants were informed about the study and had the opportunity to ask questions. Subsequently, participants signed informed consent and completed a demographic questionnaire containing background information such as sex, age, and profession. The participants did not receive any incentives for participation.

To identify potential barriers and facilitators for the implementation of the PCFU Care intervention, a semi‐structured interview guide was developed based on the theoretical framework of Grol and Wensing.^38^ This framework (Table 1) describes barriers to and incentives for change, that can influence the implementation of interventions in the medical field, at six levels of healthcare (innovation, individual professional, patient, social context, organizational context, economic and political context). The interview guide incorporated open‐ended questions on (1) current follow‐up care; (2) differences between current care and care according to the PCFU Care intervention; (3) HCPs' opinion of the PCFU Care intervention; (4) expected barriers and facilitators for implementation of the PCFU Care intervention in general and according to the six levels of Grol and Wensing and (5) needs for successful implementation of the PCFU Care intervention.

**TABLE 1:** Barriers and facilitators for implementation at different levels of healthcare; adapted from the Grol and Wensing framework.^2^

Healthcare level	Innovation	Individual professional	Patient	Social context	Organizational context	Economic and political context
Barriers and facilitators	Advantages in practice Feasibility Credibility Accessibility Attractiveness Clarity	Awareness Knowledge Attitude Motivation to change Behavioral routines Skills Feelings	Awareness Knowledge Skills Capability Attitude Compliance Motivation Feelings Behavior Preference	Collaboration Opinion of colleagues Culture of the network Leadership Environmental support	Organization of care processes Staff Capacities Resources Structures	Financial arrangements Regulations Policies

### 2.5 Data analysis

Data from focus group interviews were audio‐recorded, anonymized, transcribed verbatim, and the Italian focus group was translated into English. Three researchers coded each transcript independently. Discrepancies were discussed until a consensus was reached. A thematic analysis was performed using Atlas.ti 22.0.11 for Windows. The analysis consisted of an inductive approach followed by a deductive approach. The inductive approach started with open coding of transcripts on a sentence level. Subsequently, axial coding was used to cluster open codes into categories. The emerged categories were deductively mapped on the levels and domains within the theoretical framework of Grol and Wensing. New categories were added to the framework. Representative quotes were selected from the transcripts.

## 3 RESULTS

### 3.1 Demographics

Thirty HCPs participated in four focus groups at the LTFU care clinics for CCSs. Table 2 provides the participants' demographics. HCPs had a mean age of 51 years, 67% were female and they had 24 years of working experience on average. Group sizes within the four focus groups ranged from five to 12 participants.

**TABLE 2:** Demographics of Healthcare Providers participating in the focus groups.

Demographics of Healthcare Providers participating in the Focus Group Discussions (*n* = 30)	
Age, years mean ± SD	50.5 ± 11.4
Sex, female *n* (%)	20 (66.7%)
Occupation	
Medical Doctor	19 (65.5%)
Nurse	5 (17.2%)
Other healthcare professions (pediatric oncology counselor, psychologist, physiotherapist)	5 (17.2%)
Unknown *n* (%)	1 (3.3%)
Experience	
Working experience, years mean ± SD	23.8 ± 11.7
Working experience in long‐term follow‐up care, years mean ± SD	18.1 ± 12.7
Number of survivors seen per month during consultations	42.6 ± 45.4

### 3.2 Barriers and facilitators for implementation of the PCFU Care intervention

Tables 3 and 4 present the identified barriers and facilitators according to the six levels of Grol and Wensing. Most barriers and facilitators were identified on the organizational level. The results section elaborates on barriers and facilitators that were mentioned during at least two of the four focus group interviews. These are in bold within Tables 3 and 4.

**TABLE 3:** Expected **barriers** for the implementation of the PCFU Care intervention.

Innovation level	Individual professional level	Patient level	Social context level	Organizational level	Economic and political level
Feasibility **Possibly not feasible for survivors living far away** Uncertain whether feasible for one LTFU clinic to see total amount of survivors Attractiveness **HCPs uncertain about attractiveness of survivor questionnaire** Clarity Lack of clarity about time needed for different phases of PCFU Care intervention	Knowledge **Lack of knowledge:** **on late effects among GPs,** oncologists and adult professionals; on how to improve QoL of survivors Lack of focus on late effects of survivors Lack of knowledge among some HCPs about PCFU intervention (e.g., survivor questionnaire process) Skills **Lack of skills regarding late effects among HCPs outside LTFU care team** Motivation GPs not always motivated: to perform regular check‐ups; to refer survivors to the LTFU clinic Feelings Difficulties confronting survivors with their cancer history Feelings of uncertainty: whether LTFU care is relevant for all survivors; whether survivors want to be invited for LTFU care	Capability Lack of capability: **to complete (online) survivor questionnaire (e.g., cognitive and** digital skills); to visit LTFU clinic (physical/mental issues); to attend multiple examinations in one day due to health status Compliance Lack of compliance: **No shows** When LTFU clinic is located at treated hospital due to representing survivors' cancer history Feelings **Low trust in GPs and local care clinics** Possible feelings of fear and stress when invited to LTFU care Confrontation with ‘cancer history’ Not willing to be seen as ‘cancer patient’ Behavior Not easy to communicate about personal problems	Collaboration **Lack of collaboration with psychosocial care facilities** Lack of sharing information for treatment summary among HCPs when referring survivors to LTFU clinic Lack of shared vocabulary between HCPs difficult for making treatment summary Lack of multidisciplinary meetings to discuss follow‐up care Lack of collaboration with HCPs and care facilities outside LTFU clinic Environmental support **HCPs uncertain about availability of environmental support for survivors** (with lack of skills for questionnaire/ unable to visit LTFU clinic) Opinion of colleagues Different opinions in health departments whether LTFU care is part of oncology or internal medicine	Capacity: Time, staff, resources **Lack of time:** to prepare treatment summary; to process results of survivor questionnaire; to discuss survivor questionnaire for survivors with many problems; to support survivors' needs and guide them through phases of the PCFU Care intervention; to organize multidisciplinary meetings; to enter data of referrals and follow‐up in database **Lack of capacity to treat both acute cancer patients and survivors** **Lack of healthcare staff** (nurses, physicians, adult oncologists, psychosocial experts), administrative staff, data managers and IT experts **Lack of staff and follow‐up facilities in the future** **Lack of (access to) psychosocial care facilities** Lack of regional LTFU care facilities Lack of ICT resources: **Lack of shared electronic systems to exchange medical information** **Lack of available medical information to prepare treatment summary** Lack of available online system for survivor questionnaire Lack of available online system for sharing medical recommendations with survivors Organization of care processes Lack of systematic planning of examinations: **Organizational issues with planning multiple examinations on same day** Unable to plan follow‐up consult years in advance Unsystematic process of inviting survivors Lack of up‐to date contact information of some survivors Unclear and unsystematic process of disseminating, collecting and analyzing survivor questionnaire Difficulties managing follow‐up care for all heterogenous late effects Lack of transition process from pediatric care to adult LTFU care Structure **Lack of a psychosocial care pathway** Acute care is prioritized due to lack of separate LTFU care team Many survivors are lost to follow‐up	Financial arrangements **Convincing hospital managers to allocate resources for LTFU care is a time‐consuming process** **Lack of financial resources:** to prepare for consultation to create a platform for medical records with (automatic) survivorship data for multidisciplinary consultations to create new staff positions to extend age group for LTFU care for psychological care for social care above 18 years **Lack of sufficient financial resources for survivors:** **with lower financial means** **with long travel distances** **Lack of long‐term financial resources for sustainable implementation** No financial agreement for staff from pediatric department for survivors above 18 years Policies Part of necessary LTFU care requires referral by GP, but not all survivors are affiliated to GP Lack of involvement government to ensure equal access to care Different regions are autonomously organizing healthcare and competing for patients

*Note*: Barriers in bold were mentioned during at least two of the four focus group interviews.

**TABLE 4:** Expected **facilitators** for the implementation of the PCFU Care intervention.

Innovation level	Individual professional level	Patient level	Social context level	Organizational level	Economic and political level
Advantages in practice **Provides uniform and consistent structure for LTFU care** **Provides knowledge about LTFU care including harmonized guidelines** Considers entire cancer survivor population, **including those lost to follow‐up** **Advantages of survivor questionnaire:** Facilitates survivors to prepare consult Guides discussion about important topics for survivor Enables introvert survivors to be more open Initiates focus on psychosocial topics Provides knowledge to HCPs not involved in history of survivor Intervention personalized to survivors' needs Expands focus to address psychosocial issues Increases participation and responsibility of survivors including decision‐making autonomy Increases awareness of survivors Facilitates access to medical information for survivors Survivors are able to use survivorship care plan as reference when visiting other care facilities Survivorship care plan functions as communication tool between HCPs Involves local care facilities and GPs Facilitates stratification of survivors Attractiveness Survivor questionnaire attractive tool when well‐supported by clinic visit and good survivor‐ HCP relation Survivorship care plan attractive tool for survivors regarding knowledge about their LTFU care Credibility Innovative and high‐quality project based on well‐defined guidelines	Knowledge, awareness **Knowledge and awareness about LTFU care among important stakeholders** (i.e., GPs, local care facilities and authorities, board of physicians, HCPs from disciplines related to LTFU care) National guidelines, media interest, and conference presentations enhance stakeholders' awareness and understanding of LTFU care Informed HCPs on addressing (new) survivors' issues and medical histories Informed hospital management about PCFU Care intervention Skills **Skilled hospital staff on PCFU Care intervention** (especially survivor questionnaire) **Skilled GPs and local care facilities for LTFU** care including psychosocial issues Attitude **Positive attitude and high expectations for PCFU Care intervention** **Positive attitude towards survivor questionnaire: time saving** Motivation **High intrinsic motivation of HCPs to take care of survivors** **Motivated HCPs to convince stakeholders for LTFU care** Motivated HCPs to support survivors with survivor questionnaire	Knowledge, awareness **Awareness among survivors on importance LTFU care** Access to important information about LTFU care for survivors Motivation/compliance **Motivated survivors to complete survivor questionnaire** **Additional support for survivors lost to follow‐up/ for whom the questionnaire is demanding** Active involvement of survivors in LTFU care Survivors feel the need to be confirmed about health status Survivors appreciate receiving results and recommendations about their health Preference Survivors prefer LTFU location to be separate from treated hospital	Collaboration Collaborating with: **HCPs from different disciplines required for LTFU care** (e.g., cardiologists, oncologists, endocrinologist, gynecologist, adult healthcare specialist, psychologist, physiotherapist, occupational therapist) **GPs** **Local healthcare facilities** **Psychosocial care facilities** Pediatric hospital Cancer survivor organization Hospital management Stakeholders that can influence organizations to allocate resources **(Inter‐)national network for LTFU care strengthens argumentation for LTFU care** Collaboration structure for effective referrals between LTFU clinic and other care facilities Shared vocabulary between different specialists Cooperation for sharing IT‐systems Sharing medical results increases HCPs willingness to perform check‐ups Culture of the network Culture of trust between (local) HCPs and survivors Accountability of GPs, board of physicians and other (local) healthcare facilities Open communication and exchanging opinions about LTFU care between HCPs within and outside LTFU clinic Environmental support for survivors to complete survivor questionnaire Opinion colleagues Shared healthcare goals among HCPs from different disciplines	Capacity: Time, staff, resources Sufficient time to discuss survivor's personal issues and extend time for survivors with many problems **Sufficient staff for PCFU Care intervention:** Pediatric and adult oncologist Nurse with expertise in LTFU care HCPs from other disciplines involved in LTFU care: e.g., endocrinologists, cardiologists Psychosocial experts: psychologist, social worker, occupational physician Administrative staff: secretary, data manager/ IT‐expert **Continuously available healthcare staff and facilities as detected issues are increasing** Sufficient capacity to create LTFU care (location, polyclinics) Sustainable IT tools: **(Intelligent) IT system that is sharable between different care facilities** Database with survivors' information for treatment summary Platform for medical records with (automatic) survivorship data National registry of encountered late effects Secure IT‐system for online survivor questionnaire Website with important information about LTFU care for survivors Organization of care processes Efficient organization of survivor questionnaire process Guidance for survivors (e.g., for survivor questionnaire) Appropriate methods for promoting and distributing survivor questionnaires Reconnecting with survivors lost to follow‐up (e.g., phone contact) Opportunity for survivors to contact LTFU clinic by phone for information Predefined path and internal schedule to be able to organize multiple examinations on same day Information transfer when healthcare staff changes Transition process from pediatric to adult care Structure **Organizational structures for implementing elements of PCFU Care intervention** Opportunity for adult specialists to visit LTFU clinic to increase understanding Available health education for survivors Structure for effective referrals between LTFU clinic and other care facilities/ disciplines Organized routine for addressing medical needs with cooperating specialists from different disciplines Organizational structure to monitor and address psychosocial needs of survivors	Financial arrangements **International consortium to convince stakeholders on institutional and national level to allocate resources for LTFU care** (e.g., authorities, management of healthcare, insurance companies, hospital management) **Understanding of hospital management** about the necessary financial structure to maintain and improve LTFU care **Reported results of LTFU care** to argue for financial resources and to create more awareness **Continuous financial support and commitment** for sustainable implementation of LTFU care **Financial aid for survivors to participate in LTFU care (e.g., reimbursement for survivors living far away)** Financial arrangements for resources (e.g., staff, care facilities) Financial arrangements to bear costs of referrals in LTFU care Regulations Written agreements about responsibilities HCPs in LTFU care within different healthcare sectors Commitment from local authorities to take care of survivors (in primary and hospital care) Policies Governmental influence on equality and access to care Combined efforts (e.g., survivor organization, ministry of health) to put stress on shoulders of hospital directors to increase focus on LTFU care

*Note*: Facilitators in bold were mentioned during at least two of the four focus group interviews.

### 3.3 Innovation level: PCFU Care intervention

#### 3.3.1 Barriers

HCPs questioned the feasibility of the PCFU Care intervention for survivors with long travel distances to the LTFU clinic. Additionally, HCPs expressed uncertainty regarding the attractiveness of the survivor questionnaire, which may be impersonal, insufficient and demanding for some survivors.

That it is a minimum, the questionnaire is still a blank sheet of paper with many questions that cannot be discussed in some way, so for me this is a barrier, the questionnaire. I cannot specifically foresee this questionnaire, I fear that, compared to my experience with patients, the questionnaire is a bit impersonal, and therefore a barrier.

However, the questionnaire was also viewed as an attractive tool when well‐supported by a clinic visit and a good survivor‐HCP relation.

#### 3.3.2 Facilitators

HCPs mentioned several advantages in practice regarding the PCFU Care intervention. The intervention provides knowledge on LTFU care including evidence‐based guidelines, offers a consistent structure for addressing late effects in CCSs, and includes CCSs that are lost to follow up. Additionally, the survivor questionnaire offers practical advantages, such as aiding CCSs in preparing clinic visits and encouraging the discussion of important topics during those visits.

This [the survivor questionnaire] is to kick off the discussion […] Because if we can focus on what they have marked as “I'm very concerned” I think we meet them in the correct arena. We can start with other things, but I think by doing this we have a greater chance of hitting what they really believe is important. And we really give them a chance to think before they come to the visit, to think over their situations.

### 3.4 Professional level

#### 3.4.1 Barriers

Participants mentioned that HCPs outside the LTFU care team may lack knowledge and skills regarding LTFU care. Particularly, HCPs expected that general practitioners (GPs) lack knowledge, which might lead to ineffective referrals and follow‐up care for CCSs. Additionally, lack of training for HCPs outside the LTFU team would potentially lead to an underestimation of CCSs risks.

The training for specialists on the territory that in my opinion is absolutely lacking with particular regard to the adult world, because many are still linked to the concept of being cured and not long surviving, therefore with an underestimation of the risks of patients that in my opinion is still in place.

#### 3.4.2 Facilitators

Adequate knowledge and skills regarding LTFU care among important stakeholders, including GPs, local care facilities, and specialists from different disciplines were expected to facilitate LTFU care. In addition, participants mentioned the importance of educated hospital staff for the PCFU intervention. A team of trained staff would increase the exchange of knowledge and improves cooperation with HCPs within and outside the hospital. Another expected facilitator was the HCPs' positive attitude towards the PCFU Care intervention. The survivor questionnaire was seen as a potentially time saving tool. HCPs had high expectations and considered the intervention as a valuable innovation. They were intrinsically motivated and viewed caring for CCSs as a moral obligation. Additionally, HCPs were motivated to convince stakeholders to prioritize LTFU care, such as convincing the hospital management to allocate resources and encouraging GPs to refer survivors to the LTFU clinic.

It's a moral obligation if you treat people when they're children and adolescents that you take care of them when they're adults.

### 3.5 Patient level

#### 3.5.1 Barriers

HCPs mentioned that survivors who are dealing with complexities, such as insufficient reading skills or cognitive impairment, have difficulties or are incapable to complete the survivor questionnaire.

With brain tumor survivors, you have to pay attention. There are a couple that cannot fill in the [survivor questionnaire]. Half of them cannot fill in the questionnaire.

Besides, HCPs encountered situations where survivors did not show up or canceled their clinic visit or were unwilling to attend. Furthermore, HCPs mentioned a lack of trust among survivors towards GPs and local care facilities, which can hinder efficient organization of LTFU care.

It becomes very difficult, also because, let's face it, the trust of these patients in local centers is close to zero, especially with regard to their pathology and their previous tumor disease, etc., they don't trust anymore. So we have a lot of work to do from a cultural point of view, not only with doctors, but also with patients, and that would be a lot…

#### 3.5.2 Facilitators

Participants considered awareness of the importance of LTFU care among CCS as a facilitator for the adoption of the PCFU Care intervention. Furthermore, HCPs observed that survivors are generally motivated to respond to questionnaires.

The vast majority have filled this [the survivor questionnaire] in and bring it with them.

Providing additional support for survivors who have been lost to follow‐up in LTFU care or for those who may find the questionnaire demanding, was seen as a facilitator for their compliance with the PCFU Care intervention.

### 3.6 Social context level

#### 3.6.1 Barriers

HCPs expressed concerns about the anticipated lack of collaboration with psychosocial care facilities.

The connection with the territorial facilities on these mental and psychosocial health aspects is absolutely null, because these are particular patients who sometimes do not have pure psychiatric disorders, but have reactive syndromes, rather than from employment, social, economic, sentimental point of view, they are behind, and no one takes charge of these needs.

This means that survivors' psychosocial needs may not be adequately addressed, despite the high demand for psychosocial care among the survivor population. Another potential barrier mentioned by HCPs was the uncertainty whether survivors who face difficulties with completing the survivor questionnaire or attending the LTFU clinic, have the opportunity to receive environmental/family support to assist them.

#### 3.6.2 Facilitators

HCPs expected that collaborations with various stakeholders in the context of LTFU care would facilitate the organization of LTFU care. This collaborative effort would involve cooperation with GPs, local care facilities, HCPs from different disciplines, psychosocial care facilities and the (inter)national network regarding LTFU care. These collaborations would facilitate the establishment of a care pathway for survivors, effective referrals and communication across healthcare disciplines, appropriately sharing of medical information, addressing psychosocial needs, and raising awareness about LTFU care.

There is plenty of collaboration from all the specialists, we are organised for the most serious complications, and we have good cooperation in such different fields even with adult experts.

Having such prominent collaborators like cardiologists here is another facilitator. Because we need to work on different levels to have internal medicine people who will focus on […], for example cardiovascular risk. That's something we have the common goals, like oncologists and cardiologists because we foresee some troubles in our survivors being 50 years old.

### 3.7 Organizational level

#### 3.7.1 Barriers

HCPs mentioned a lack of time and staff for various components of the PCFU Care intervention. Specifically, time limitations were expected for the survivor questionnaire procedures, such as processing the questionnaire results before the clinic visits. Moreover, the treatment summary was seen as a time‐consuming process. HCPs also indicated the challenge to both treat acute cancer patients and take care of survivors in terms of available capacity. Due to limited capacity, acute care is often prioritized over LTFU care. In the future, an increasing number of survivors will be seen at the LTFU clinic. The lack of staff, resources, and care facilities were major barriers for sustainable implementation.

HCPs mentioned that the lack of available medical data and efficient Information and Communication (ICT) support would hinder the effective exchange of medical information for the treatment summary.

We have different types of patient records, so, in the most of our area we can read the results, it is just one same system, but then there are also three more that are totally different and don't communicate. So, you rely on papers and papers being scanned and so. That is how the systems are, there are different healthcare providers who don't really speak electronically to each other. So if I would point out a possible barrier then it is still the communication with the three other regions which work with different communications systems and send you the patient data if they send it to you at all via paper or via post mail.

Besides, HCPs would face difficulties with organizing multiple examinations on the same day. Another barrier was the lack of available psychosocial support and the absence of a structured psychosocial care pathway to adequately address survivors' psychosocial needs.

We prevent the second tumor, we make them responsible for their project of care and life, but then instead all the psychosocial aspects that we identify, a treatment has not been thought through. On the field there are no structures to welcome them, there are no paths, there is no specific training, because either they are placed in the melting pot of adult psychiatric patients, and there is no response to their needs, or the problem is underestimated, and then they are left to themselves.

#### 3.7.2 Facilitators

HCPs identified specific professional roles that will facilitate the implementation of the PCFU Care intervention, as outlined in Table 3 under organizational level within the subdomain ‘capacity.’ The nurse plays an important role in the guidance of survivors through the PCFU Care intervention. When CCSs receive guidance, HCPs expected that questionnaire responses will improve. Data‐managers and Information Technology (IT) experts would facilitate the extraction of data for treatment summaries and the development of high‐quality IT‐systems that are shareable between different healthcare settings to exchange medical information. Psychosocial roles, including psychologists, social workers, and occupational physicians were seen as important to guide CCSs from a psychosocial perspective. Having staff continuously available would facilitate sustainable implementation.

We're recently well‐staffed for doctors, I would say. There are four at least at the department of oncology and two in the department of pediatric oncology.

We have excellent secretaries that support us in such a good way.

We have since two, three years now [an occupational therapist].

I think it's optimal. If you can have somebody [a nurse] but with the skills. It's not any nurse. It's a nurse with the skills. I mean, you can train somebody, but [our nurse] comes with the knowledge of […] having worked with these patients already at the department of endocrinology on all the late effects associated there. So, we were blessed to have somebody who comes fully equipped from the beginning.

Furthermore, the alignment of organizational structures and operating procedures with the elements of the PCFU Care intervention would facilitate its implementation. An organized structure would support efficient management of medical and psychosocial needs for CCSs.

### 3.8 Economic and political level

#### 3.8.1 Barriers

HCPs reported that convincing hospital managers to prioritize LTFU care and allocate adequate resources is a time‐consuming process. Insufficient funding for certain components of the PCFU Care intervention and limitations in financing psychosocial care were identified. Furthermore, the lack of sufficient financial support for survivors might prevent them from attending the LTFU clinic. Some survivors would be willing to return to the LTFU clinic, but their financial situation would hinder them from coming to the LTFU clinic. Survivors with long travel distances face travel and accommodation costs and lost days of work that are usually not reimbursed.

We have patients who also come from outside the region, therefore they have to face expenses both in terms of travel and stay in the hospital facilities, therefore high costs that are not always reimbursable, and so also then especially with regard to the group of adults and young adults, even lost days of work, so this type of organization it is a barrier.

HCPs mentioned the uncertainty of long‐term financial resources for LTFU care as a barrier to achieve sustainable implementation.

#### 3.8.2 Facilitators

HCPs mentioned several facilitators for achieving (long‐term) financial resources for LTFU care. International cooperation within LTFU care for CCSs would strengthen the argumentation for convincing stakeholders at both institutional and national levels to allocate resources for LTFU care. Reporting results and examples of LTFU care would raise awareness and advocates for sustainable resource allocation.

Hammering on the authorities to make them understand that this is an issue. And there it's always good to have the numbers. It's always good to have the numbers to be able to say, There are this many people, they are these ages, they have such and such issues, we see them at this and that regularity. And I think the only thing that will affect someone sitting on the money and on the resource is being convinced by numbers, by data. Yeah, and try to calculate the health economics about it.

Additionally, financial aid for survivors was expected to facilitate their participation in LTFU care.

## 4 DISCUSSION

This study presents the first qualitative pre‐implementation study exploring barriers and facilitators for implementing high‐quality LTFU care for CCSs from the HCPs' perspective in four LTFU care clinics in Europe. Barriers and facilitators were identified within all six levels of the Grol and Wensing framework. Most barriers were identified on the organizational level, including insufficient staff, time, capacity, and psychosocial support. Other main barriers included limited knowledge of late effects among HCPs outside the LTFU care team, inability of some survivors to complete the survivor questionnaire and lack of (long‐term) financial resources. Main facilitators included motivated HCPs and survivors, skilled hospital team, collaborations with important stakeholders like GPs and psychosocial care facilities, utilization of the international collaboration and reporting LTFU care results to convince hospital managers.

The potential implementation challenges lack of time, staff, and (ICT) resources have been previously mentioned by HCPs for implementing LTFU care.^33^, ^40^, ^41^ As potential implementation strategies to mitigate these barriers, establishing efficient organizational structures that incorporate collaborative ICT systems (e.g., automatic data generation from electronic medical records, treatment summary databases, web‐based survivor care plans) could be considered.^31^, ^41^, ^42^, ^43^ In addition, contracting specialized survivorship nurses, administrative staff, and data managers could be an implementation strategy to alleviate the oncologist's workload.^41^ Collaboration with hospital management and health insurers is crucial for resource allocation. Demonstrating results and cost‐effectiveness of LTFU care can support resource allocation at institutional and national levels.

Another main study result is the need to enhance knowledge and collaboration with GPs and HCPs from various disciplines and healthcare facilities. This can facilitate effective exchange of medical information between HCPs, appropriate CCSs referrals, and the establishment of suitable care pathways to address CCSs' physical and psychosocial needs. The importance of improving knowledge, communication and collaboration aligns with previous literature,^33^, ^34^, ^40^, ^41^ which primarily focused on LTFU shared‐care models. Professional education for HCPs, including GPs, could be an implementation strategy to improve competence in LTFU care.^34^, ^44^ Our study suggests that survivors might lack trust in GPs and local healthcare facilities regarding LTFU care, potentially lowering compliance. HCP education has the potential to increase survivor's confidence in HCPs and GPs competencies as well.^44^

The present study highlights a gap in detecting psychosocial issues among CCSs at the LTFU clinic and the absence of an adequate follow‐up care pathway to manage these issues. It is essential to address this barrier as psychosocial issues are commonly experienced by survivors.^15^, ^45^, ^46^ The importance of addressing CCSs psychosocial needs is underlined by the Institute of Medicine.^47^ It is crucial to improve access to and collaboration with psychologists, and social workers along with the establishment of a referral structure for psychosocial care.

Our study also showed that some survivors may face limitations in participating in the PCFU Care intervention due to physical, mental, financial, and logistical challenges. Therefore, as an implementation strategy providing guidance, such as aiding survivors with completing the survivor questionnaire, could be considered. Additionally, financial reimbursements as implementation strategies can aid survivors who face financial difficulties or who must cover travel and accommodation costs. However, dedicated funding to address the cost and travel burden for survivors remains a challenge.^41^ Online consultations and interventions may be a viable alternative for survivors who cannot easily visit the LTFU clinic.

Prior reviews on implementing LTFU care for cancer survivors have predominantly examined GP‐led LTFU care models, shared care models between GP and cancer specialists and oncology nurse‐led LTFU care models, with a focus on adult‐onset cancers.^33^, ^34^ The strength of our study is that it concentrates on establishing LTFU clinics as care model for the heterogenous CCS population. LTFU clinics have the capability to manage CCSs who require complex care due to elevated risks of serious late effects.^25^, ^26^, ^48^ Another strength of this study lies in its incorporation of insights from diverse European healthcare systems, providing practical and detailed information on important barriers to address and facilitators to use for successful implementation efforts. This diverse overview of barriers and facilitators based on real‐world settings is relevant for other hospitals willing to implement LTFU care for CCSs. Findings can be integrated in implementation strategies to enhance the provision of LTFU care for CCSs in Europe.

A limitation is that this study only considers the HCPs perspective. To design a comprehensive implementation strategy, future research should include perspectives from CCSs and their informal caregivers, hospital management, and policy makers. Another limitation is that data saturation may not have been fully reached in the four focus groups. Determining data saturation becomes more challenging when utilizing focus groups. Some barriers and facilitators were saturated across all four focus groups while other factors were more specific to particular clinic sites. These contextual variations should be taken into account when designing a fitted implementation strategy for LTFU care. However, this study aimed to explore barriers and facilitators proposed by a varied group of HCPs from different European healthcare systems, with the purpose of gathering a diverse overview of barriers and facilitators. This study included 30 participants, among whom the most important HCPs involved in LTFU from the four European clinics that are part of the PCFU study. This exploratory design has the advantage of being relatively fast, inexpensive and can be replicated by other centers to identify barriers and facilitators specific to their healthcare setting with minimal resources.

When interpreting results, cultural differences may affect expressed content and openness in focus groups. Additionally, two centers used their native language during the focus groups and the other two used the English language, which could have influenced the level of participation. However, centers could choose their preferred language, assuming English proficiency when opting for English. In Italy, the HCPs preferred to conduct the focus group in Italian, which was then translated into English one‐way. Unfortunately, there's a potential risk of losing meaning by not translating the English version back into Italian.

## 5 CONCLUSION

This study identified expected barriers and facilitators from the HCPs' perspective for successful implementation of high‐quality LTFU care for CCSs using the PCFU Care intervention. Our findings showed that specific attention should be given to knowledge, capacity, and financial issues, along with addressing psychosocial issues of survivors. The results support clinical staff in providing optimal LTFU care and offer practical guidance for integrating the PCFU Care intervention.

## AUTHOR CONTRIBUTIONS

**Dionne Breij:** Conceptualization (equal); data curation (lead); formal analysis (lead); investigation (equal); methodology (equal); project administration (equal); writing – original draft (lead). **Lars Hjorth:** Funding acquisition (equal); resources (equal); writing – review and editing (equal). **Eline Bouwman:** Conceptualization (equal); data curation (equal); methodology (equal); writing – review and editing (equal). **Iris Walraven:** Supervision (lead); writing – review and editing (lead). **Tomas Kepak:** Funding acquisition (equal); resources (equal); writing – review and editing (equal). **Katerina Kepakova:** Project administration (equal); resources (equal); writing – review and editing (equal). **Riccardo Haupt:** Funding acquisition (equal); resources (equal); writing – review and editing (equal). **Monica Muraca:** Funding acquisition (equal); project administration (equal); resources (equal); writing – review and editing (equal). **Irene Göttgens:** Conceptualization (equal); data curation (lead); formal analysis (lead); investigation (equal); methodology (equal); project administration (equal); writing – review and editing (equal). **Iridi Stollman:** Data curation (equal); formal analysis (lead); writing – review and editing (supporting). **Jeanette Falck Winther:** Funding acquisition (equal); writing – review and editing (equal). **Anita Kienesberger:** Writing – review and editing (supporting). **Hannah Gsell:** Writing – review and editing (supporting). **Gisela Michel:** Writing – review and editing (equal). **Nicole Blijlevens:** Supervision (supporting); writing – review and editing (supporting). **Saskia M. F. Pluijm:** Funding acquisition (equal); writing – review and editing (equal). **Katharina Roser:** Writing – review and editing (equal). **Roderick Skinner:** Funding acquisition (equal); writing – review and editing (equal). **Marleen Renard:** Writing – review and editing (supporting). **Anne Uyttebroeck:** Funding acquisition (equal); resources (equal); writing – review and editing (equal). **Cecilia Follin:** Project administration (equal); resources (equal); writing – review and editing (supporting). **Helena J. H. van der Pal:** Funding acquisition (equal); writing – review and editing (equal). **Leontien C. M. Kremer:** Conceptualization (equal); funding acquisition (lead); methodology (equal); writing – review and editing (equal). **Jaqueline Loonen:** Conceptualization (lead); funding acquisition (lead); methodology (equal); supervision (lead); validation (equal); writing – review and editing (lead). **Rosella Hermens:** Conceptualization (lead); funding acquisition (lead); investigation (equal); methodology (lead); project administration (supporting); supervision (lead); validation (equal); writing – review and editing (lead).

## FUNDING INFORMATION

“The project has received funding from the European Union's Horizon 2020 research and innovation program under grant agreement No 824982. The material presented and views expressed here are the responsibility of the author(s) only. The EU Commission takes no responsibility for any use made of the information set out.”

## CONFLICT OF INTEREST STATEMENT

The authors declare that they have no known competing financial interests or personal relationships that could have appeared to influence the work reported in this paper.

## ETHICS STATEMENT

This study adhered to local (METC) procedures of the participating centers. The full names of the ethics committees were: Ethische Commissie Onderzoek UZ/KU Leuven (S63072). Facultni nemocnice u sv. Anny v Brno, Eticka komise (41 V/2019). According to national legislation and confirmed by the Health directors of the participating institutes, no ethical approval was needed in Lund and Italy.

## CONSENT

Informed consent was obtained from all participants.

## PERMISSION TO REPRODUCE MATERIAL FROM OTHER SOURCES

Permission via Rightslink Elsevier to reuse a figure that is previously published in The European Journal of Cancer: van Kalsbeek RJ, Mulder RL, Haupt R, Muraca M, Hjorth L, Follin C, et al. The PanCareFollowUp Care Intervention: A European harmonized approach to person‐centred guideline‐based survivorship care after childhood, adolescent and young adult cancer. *European Journal of Cancer*. 2022;162:34–44 Figure 1. The PanCareFollowUp Care Intervention steps: previsit preparation, clinic visit, and follow‐up call.

## Data Availability

The PanCareFollowUp project aims to comply with all the four FAIR principles and to share individual de‐identified data upon request.
